# Saffron (*Crocus sativus*) versus duloxetine for treatment of patients with fibromyalgia: A randomized double-blind clinical trial

**Published:** 2018

**Authors:** Mansoor Shakiba, Ehsan Moazen-Zadeh, Ahmad Ali Noorbala, Morteza Jafarinia, Parisa Divsalar, Ladan Kashani, Nazila Shahmansouri, Abbas Tafakhori, Hannaneh Bayat, Shahin Akhondzadeh

**Affiliations:** 1 Psychosomatic Research Center, Imam Hospital, Tehran University of Medical Sciences, Tehran, Iran; 2 Psychiatric Research Center, Roozbeh Hospital, Tehran University of Medical Sciences, Tehran, Iran; 3 Infertility Ward, Arash Hospital, Tehran University of Medical Sciences, Tehran, Iran; 4 Neurology Ward, Imam Hospital, Tehran University of Medical Sciences, Tehran, Iran; † Equal first author

**Keywords:** Crocus sativus, Depression, Duloxetine, Fibromyalgia, Saffron

## Abstract

**Objective:**

Saffron was found efficient and safe in treatment of neuropsychiatric disorders, in particular depression. We compared the efficacy of saffron with duloxetine in treatment of patients with fibromyalgia.

**Materials and Methods:**

In this double-blind parallel-group clinical trial, outpatients with fibromyalgia were randomized to receive either saffron 15 mg or duloxetine 30 mg starting with 1 capsule per day in the first week followed by 2 capsules per day from week 2 until the end of week 8. Participants were men and women aged 18-60 years diagnosed with fibromyalgia based on the American College of Rheumatology 2010 criteria who also had a pain score≥40 based on visual analogue scale. Participants were excluded in case they had rheumatologic diseases, inflammatory/infectious/autoimmune arthritis, comorbid neuropsychiatric disorders except depressive disorders, pain due to traumatic injuries, drug history of duloxetine or saffron use, current use of psychoactive medications, recent use of muscle relaxants, steroids, opioid analgesics, benzodiazepines, anti-epileptics, or injective analgesics. Primary outcomes included differences in mean score changes from baseline to endpoint between the treatment arms for Hamilton Rating Scale for Depression, Fibromyalgia Impact Questionnaire, and Brief Pain Inventory.

**Results:**

Socio-demographic characteristics and baseline scores were similarly distributed between the two treatment arms (2n=46). No significant difference was detected for any of the scales neither in terms of score changes from baseline to endpoint between the two treatment arms (Mean score changes: -4.26 to 2.37; p-values: 0.182-0.900) nor in terms of timetreatment interactions (p-values: 0.209-0.964).

**Conclusions:**

Saffron and duloxetine demonstrated comparable efficacy in treatment of fibromyalgia symptoms.

## Introduction

As a chronic debilitating disorder with challenging diagnosis and treatment, fibromyalgia (FM) affects 1-10% of general population (Queiroz, 2013 ▶), mostly women; FM is defined by chronic widespread pain usually accompanied by other symptoms including anxiety/depressive symptoms, fatigue, sleep disturbances, cognitive dysfunction, and headache as well as other somatic symptoms (Schmidt-Wilcke and Clauw, 2011 ▶). While absence of organic abnormalities gives a benign appearance to FM, the real “iceberg–like” burden of the disorder is estimated to be comparable to those of diabetes and hypertension (Ghavidel-Parsa et al., 2015 ▶). 

Mental symptoms of FM are so prominent and interwoven with pain that there was a debate on classification of FM as a somatoform disorder (Häuser and Henningsen, 2014a ▶). Major depressive disorder (MDD) is highly prevalent in patients with FM, affecting 22-90% of these individuals (Pae et al., 2008 ▶; Gracely et al., 2012 ▶). Besides, FM overlaps MDD in many aberrant neurobiological findings. Decreased antioxidant capacity and increased oxidative stress are well documented in both FM and MDD (Cordero et al., 2010 ▶; Meeus et al., 2013 ▶). Decreased serotonin levels and alterations in the serotonergic system are supposed to be the major culprit in MDD. Moreover, lowered levels of serotonin and norepinephrine are implicated in the pathophysiology of chronic pain and mental symptoms of FM (Maletic and Raison, 2009 ▶; Becker and Schweinhardt, 2012 ▶; Sluka and Clauw, 2016 ▶). Furthermore, it is suggested that both conditions share genetic and environmental risk factors (Pae et al., 2008 ▶; Gracely et al., 2012 ▶), including various psychological or biological stressors (Schmidt-Wilcke and Clauw, 2011 ▶; Becker and Schweinhardt, 2012 ▶; Thiagarajah et al., 2014 ▶). 

Aside from several non-pharmacologic approaches, more than 40 compounds were investigated for treatment of FM, among which antidepressants are routinely prescribed to these patients (Calandre et al., 2015 ▶; Chinn et al., 2016 ▶). Meanwhile, FDA approved only three compounds, including duloxetine, milnacipran, and pregabalin, and Canada Health approved only duloxetine and pregabalin for treatment of fibromyalgia. Duloxetine is a norepinephrine serotonin reuptake inhibitor (NSRI), and pregabalin alters voltage-gated calcium channel function (Chinn et al., 2016 ▶). According to comprehensive reviews, duloxetine mainly improves pain and depressive symptoms of FM with minimal effects on fatigue and sleep disturbances (Häuser et al., 2013 ▶), while pregabalin results in pain reduction and sleep disturbances with no effect on fatigue (Üçeyler et al., 2013 ▶) and less influence on depression compared to duloxetine (Häuser et al., 2010 ▶; Calandre et al., 2015 ▶). 

Despite improvements experienced by some patients with FM following treatment with the approved medications, the symptoms tend to be intractable in most patients and these patients have to repeatedly attend specialty clinics (Ghavidel-Parsa et al., 2015 ▶; Theoharides et al., 2015 ▶). Also, it is argued that beneficial effects of approved medications on pain only minimally outweigh their side effects, and although the emergent adverse events are not usually serious, rates of adherence to treatment are low due to tolerability issues (Häuser et al., 2014b ▶). Therefore, seeking for novel therapeutics are underway (Ablin and Häuser, 2016 ▶; Lawson, 2016 ▶). Among them, complementary and alternative approaches such as phytotherapy have yielded promising results (de Souza Nascimento et al., 2013 ▶; Lauche et al., 2015 ▶). Saffron (*Crocus sativus* L.) has appeared as a novel interesting candidate for treatment of FM since its benefits in several medical conditions, including neuropsychiatric disorders, are well documented (Moshiri et al., 2015 ▶). In particular, saffron was found beneficial in clinical trials of depressive disorders (Hausenblas et al., 2013 ▶; Lopresti and Drummond, 2014 ▶; Mazidi et al., 2016 ▶; Kashani et al., 2018 ▶) and anxiety (Ghajar et al., 2016 ▶; Mazidi et al., 2016 ▶), as well as animal models of chronic pain (Milajerdi et al., 2015 ▶; Safakhah et al., 2016 ▶; Amin et al., 2017 ▶). More interestingly, saffron efficacy was comparable to those of citalopram and fluoxetine, as two selective serotonin reuptake inhibitors (SSRIs) (Noorbala et al., 2005 ▶; Basti et al., 2007 ▶; Shahmansouri et al., 2014 ▶; Ghajar et al., 2016 ▶; Kashani et al., 2017 ▶), and to imipramine as a tricyclic antidepressant (TCA) with inhibitory effects on both serotonin and norepinephrine reuptake (Akhondzadeh et al., 2004 ▶). The antidepressant activity of saffron is attributed to several biological mechanisms (Lopresti and Drummond, 2014 ▶). In fact, compared to the routine antidepressants including SSRIs, NSRIs, and TCAs, saffron is interesting not only for its effects on neurotransmitters, but also for its potent antioxidant activity as well as neuroprotective and anti-inflammatory effects (Lopresti and Drummond, 2014 ▶; Khazdair et al., 2015 ▶), all of which make it a potential candidate for treatment of FM. Furthermore, saffron has an excellent safety profile with few tolerable side effects (Moshiri et al., 2015 ▶), and beyond that, it was beneficial in treatment of sexual dysfunction as a frequent side effect of both SSRIs and NSRIs (Modabbernia et al., 2012 ▶; Kashani et al., 2013 ▶).

Based on mentioned grounds, in this randomized double-blind clinical trial, we compared the efficacy of saffron and duloxetine in treatment of fibromyalgia symptoms by defining primary outcomes as differences in change of scores from baseline to endpoint between the two treatment arms using the Hamilton Rating Scale for Depression (HRSD), Fibromyalgia Impact Questionnaire (FIQ), and Brief Pain Inventory (BPI). 

## Materials and Methods

**Trial design and setting**

In this double-blind clinical trial, patients with FM were randomized to receive either saffron or duloxetine for 8 weeks at the outpatient rheumatology clinic, Imam Khomeini Hospital, Tehran, Iran, from September 2016 to April 2017. Institutional Review Board (IRB) of Tehran University of Medical Sciences approved the study according to the World Medical Association’s code of ethics (Declaration of Helsinki, revised in Brazil 2013), and the study was registered at the Iranian Registry of Clinical Trials (IRCT201604261556N91; www.irct.ir). All patients and their caregivers provided written consent after being clearly informed that they were free to withdraw from the study at any time without affecting their relationship with their healthcare provider. 

**Participants**

Participants were men and women aged 18-60 years who were diagnosed with FM based on the American College of Rheumatology (ACR) 2010 criteria (Wolfe et al., 2010) and had a pain score of over 40 out of 100 based on visual analogue scale (VAS). 

In case the candidates met any of the following conditions, they were excluded from the study: comorbid neuropsychiatric disorders other than depressive disorders based on the Diagnostic and Statistical Manual of Mental Disorders-IV-Text Revision (DSM-IV-TR) (American Psychiatric Association, 2000 ▶); substance use disorder during the 2 years prior to the study; suicidal ideation; rheumatologic diseases other than FM; inflammatory, infectious , or autoimmune arthritis; multiple sclerosis; pain due to traumatic injuries; serious medical conditions; history of multiple surgeries; pregnancy, breast feeding, or women with no contraception history; history of treatment with duloxetine or saffron; current use of psychoactive medications specially serotonergic compounds or MAO-Is; use of muscle relaxants, steroids, opioid analgesics, benzodiazepines, or anti-epileptics during the 1 week prior to the study based on detailed history taking; injection of analgesics to painful areas during the 1 month prior to the study; history of using thioridazine, acetylcholinesterase inhibitors, warfarin, or medications affecting the P450 CYP4A3 enzyme in the 2 weeks before the study; and history of hypersensitivity to herbal compounds, in particular saffron.

**Intervention**

Saffron (IMPIRAN, Iran) and duloxetine capsules (Sobhan Darou, Iran) contain 15 and 30 mg respectively. Patients were randomized to receive either saffron or duloxetine starting with 1 capsule per day in the first week followed by 2 capsules per day from the second week till the eighth week.

Capsule counts and reports by participants were used to check for adherence to treatment. Participants were asked not to use muscle relaxants, steroids, opioid analgesics, or benzodiazepines during the trial on a routine basis; however, occasional use of over the counter medications was allowed. 

**Figure 1:**
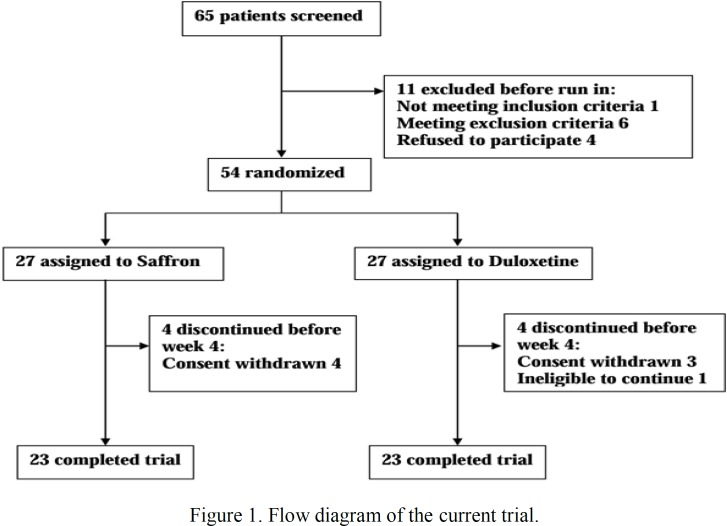
Flow diagram of the current trial

**Preparation of saffron capsule**

A closely monitored protocol was adopted. First, 1800 mL of ethanol (80%) was used to process 120 g of dried and milled *Crocus sativus* L. stigma through a three-step percolation procedure. Dried saffron extract was prepared by evaporating ethanol at 35-40 °C; final capsules contained 15 mg of saffron extract (crocin 1.65-1.75 mg), sodium starch glycolate (disintegrant), magnesium stearate (lubricant), and lactose (filler). Capsules were standardized based on crocin content by evaluating the concentration of major saffron constituents, crocin and safranal, using spectrophotometry with direct absorbance readings of 440 and 330 nm, respectively. 

**Outcomes**

Primary outcomes included differences in mean change from baseline to endpoint between the treatment arms for the Hamilton Rating Scale for Depression (HRSD) score (Hamilton, 1960 ▶), Fibromyalgia Impact Questionnaire (FIQ) total score (Burckhardt et al., 1991 ▶), and the Brief Pain Inventory (BPI) pain score (average of 4 subscales) (Cleeland and Ryan, 1994 ▶). Secondary outcomes were defined as difference in mean change of scores from baseline to the endpoint between the treatment arms for pain Visual Analogue Scale (VAS) (Carlsson, 1983 ▶), Multi-dimensional assessment of fatigue (Hewlett et al., 2011 ▶), and Hospital Anxiety and Depression (HADS) subscales (Zigmond and Snaith, 1983 ▶). Also, time treatment interaction effect was assessed for all the scales. The mentioned scales were recruited previously in Iranian clinical studies (Sadreddini et al., 2008 ▶; Ghavidel-Parsa et al., 2014 ▶; Moazen-Zadeh et al., 2018 ▶). All measurements were performed by two experienced raters with inter-rater reliability of >90%.

**Safety and adverse events**

All participants were subjected to thorough clinical examinations and paraclinical evaluations before entering the study. After the baseline visit, patients were visited at week 1 with a focus on emergent adverse events using a 25-item comprehensive checklist (Asadabadi et al 2013; Rezaei et al., 2013 ▶; Khodaie-Ardakani et al., 2014 ▶). Also, it was required that both patients and their caregivers report any unexpected signs and symptoms during the trial. At week 4, thorough clinical examinations and lab-tests were performed again. An independent psychiatrist and a rheumatologist were responsible for making decisions on medication continuation, discontinuation, or dose adjustment in case of adverse events.

**Sample size**

Published trials concerning administration of duloxetine in FM, as well as expert clinician opinion, were used as the basis for determining effect size for sample size estimation. We estimated 3 sample sizes based on the 3 primary outcomes and chose the biggest one for this study. In details, a mean difference in HRSD score change of 2, a standard deviation of 2.5, a type I error of 5%, and a type II error of 20% estimated a primary sample size of 45. Considering an attrition rate of 20%, the final sample size was calculated as 54 for total number of patients in both arms. 

**Randomization, allocation concealment, and blinding**

Randomization to either saffron or the duloxetine arm, was carried out in a 1:1 ratio through computerized random number generation by an independent person. Treatment allocation concealment was achieved using sequentially numbered sealed opaque envelopes. Saffron capsules were identical to duloxetine in shape, size, texture, odor, and color. Medications were distributed by an independent investigational drug pharmacist. Both healthcare providers and participants were blinded. 

**Statistical analysis**

IBM SPSS Statistics 19.0.0 (IBM Corporations) was the software of choice for analysis. Measures of central tendency included number (%), mean (SD or SE), and mean (95% confidence intervals (95% CI)). The last observation carried forward (LOCF) method was used for intention-to-treat (ITT) analysis. Means were compared using the independent samples t-test, and if Levene’s test indicated violation of equality of variances assumption, degrees of freedom and p-values were corrected. Frequencies were compared using Fisher’s exact test or its Freeman-Halton extension. Timetreatment interactions were estimated through a two-way repeated measures analysis of variance (ANOVA) and in case of violation of sphericity assumption, Greenhouse-Geisser correction was applied. Significance level was set at 0.05 in all analyses considering two-sided p-values.

## Results

**Participants and baseline characteristics**

Sixty-five patients were screened but 54 were randomized to saffron and duloxetine treatment arms equally (Figure. 1). In each treatment arm, 23 participants had the first post-baseline visit and completed the trial. There were no significant differences in socio-demographic characteristics, except for marital status, between the two treatment arms (Table 1).

**Table 1:** Baseline characteristics of patients with fibromyalgia

	**Mean (SD) or N (%)**		**P-value **
	**Saffron (n=23)**	**Duloxetine (n=23)**	
**Age, years**		42.35 (9.83)	41.61 (9.14)	0.793
**Female Gender, n**		18 (78.26)	16 (69.57)	0.738
**Marital Status, n**				0.028
**Single**		6 (26.09)	1 (4.35)	
**Married **		16 (69.56)	22 (95.65)	
**Divorced**		1 (4.35)	0 (0)	
**Level of Education, n**				0.506
**Primary school**		9 (39.13)	7 (30.43)	
**Secondary school/ diploma**		4 (17.39)	2 (8.70)	
**University degree**		10 (43.48)	14 (60.87)	

**Outcomes**

There was no significant difference in baseline scores between the two treatment arms for any of the scales (i.e. HRSD, FIQ, BPI, VAS, GFI, HADS-Depression, or HADS-Anxiety scores) (Table 2). All scale scores decreased in both arms during the trial as a response to treatment. Meanwhile, neither primary outcome nor secondary outcomes varied significantly. In other words, no significant difference was detected for any of the scales neither in terms of score changes from baseline to endpoint between the two treatment arms (Mean score changes: -4.26 to 2.37; p-values: 0.182-0.900) nor in terms of timetreatment interactions (p-values: 0.209-0.964) (Table 2). Further analysis did not reveal any sensible change to the results using the marital status as the covariate.

**Table 2:** Change in scales’ score from baseline in patients with fibromyalgia treated with saffron (n=23) or duloxetine (n=23).

	**Mean (SD)**			**Mean (SE)**	**Mean (CI 95%)**	**Independent samples t-test**		**Two-way repeated measures ANOVA**	**p-value**
	**Week 0**	**Week 4**	**Week 8**	**Baseline to endpoint**	**Difference in change**	**t (df)**	**p-value**	**F (df, mean square)**	
**HRSD**									
**Saffron**	14.83 (4.86)	11.41 (5.17)	8.96 (3.48)	-5.87 (0.77)	0.16 (-2.39 to 2.71)	0.13 (44)	0.900	0.12 (2, 1.01)	0.888
**Duloxetine**	14.74 (4.69)	10.75 (4.56)	8.71 (3.95)	-6.03 (1.00)					
**FIQ**									
**Saffron**	52.65 (13.34)	47.62 (10.61)	42.13 (10.75)	-10.53 (1.94)	2.37 (-3.73 to 8.46)	0.78 (44)	0.438	0.58 (1.55, 30.31)	0.520
**Duloxetine**	54.25 (11.87)	46.64 (10.52)	41.35 (10.48)	-12.90 (2.32)					
**BPI**									
**Saffron**	7.88 (1.02)	7.46 (1.19)	5.67 (1.63)	-2.21 (0.29)	-0.36 (-1.41 to 0.69)	-0.69 (38.35)	0.493	0.41 (2, 0.62)	0.667
**Duloxetine**	8.09 (1.62)	7.59 (1.96)	6.24 (2.21)	-1.85 (0.43)					
**VAS**									
**Saffron**	60.43 (15.51)	40.87 (16.21)	38.48 (16.20)	-21.96 (3.51)	-4.26 (-15.64 to 7.12)	-0.76 (44)	0.454	0.46 (1.39, 81.29)	0.564
**Duloxetine**	58.13 (24.39)	41.74 (12.30)	40.43 (12.61)	-17.70 (4.42)					
**GFI**									
**Saffron**	42.03 (8.62)	31.26 (8.00)	25.13 (9.63)	-16.90 (2.40)	0.68 (-6.52 to 7.87)	0.19 (44)	0.850	0.04 (2, 2.19)	0.964
**Duloxetine**	41.51 (8.37)	29.93 (6.42)	23.93 (8.36)	-17.58 (2.64)					
**HADS: Depression**									
**Saffron**	12.52 (3.44)	11.52 (2.81)	9.22 (3.59)	-3.30 (0.88)	1.74 (-0.85 to 4.34)	1.36 (44)	0.182	1.63 (1.39, 14.39)	0.209
**Duloxetine**	13.35 (3.31)	10.91 (2.43)	8.30 (2.79)	-5.04 (0.94)					
**HADS: Anxiety**									
**Saffron**	14.70 (4.09)	13.00 (3.36)	11.13 (3.32)	-3.57 (0.56)	0.71 (-0.95 to 2.37)	0.86 (44)	0.395	0.53 (2, 1.64)	0.591
**Duloxetine**	14.53 (3.73)	12.26 (3.32)	10.26 (2.77)	-4.27 (0.60)					

HRSD, Hamilton Rating Scale for Depression; FIQ, Fibromyalgia Impact Questionnaire; BPI, Brief Pain Inventory; VAS, Visual Analogue Scale; GFI, Global Fatigue Index; and HADS, Hospital Anxiety and Depression Scale.

**Adverse Events**

No significant difference was detected between the two arms for the adverse events that were at least reported once by a patient in the total sample size (Table 3).

**Table 3:** Incidence of adverse events in patients with fibromyalgia

	**Number (%) of Patients**		**P-value**
**Adverse event**	**Saffron** **(n=23)**	**Duloxetine** **(n=23)**	
**Abdominal pain**	1 (4.35)	0 (0)	1.000
**Nausea**	2 (8.70)	4 (17.39)	0.665
**Diarrhea**	1 (4.35)	0 (0)	1.000
**Decreased appetite**	0 (0)	3 (13.04)	0.233
**Head ache**	0 (0)	2 (8.70)	0.489

## Discussion

Novel treatments for FM are being actively investigated with a few drugs approved and other potential therapeutics being under clinical investigation. While duloxetine is an approved medication for treatment of FM, limited efficacy and low adherence have been reported in previous trials. Saffron showed similar efficacy to SSRIs and TCAs for treatment of depression, and has an excellent safety profile as reported by previous studies. In this clinical trial, patients with FM experienced comparable improvement in symptoms in both saffron and duloxetine arms based on HRSD, FIQ, BPI, GFI, VAS pain, HADS- depression, and HADS- anxiety scores.

To the best of our knowledge, this is the first report on administration of saffron to patients with FM and the first clinical trial to compare the efficacy of saffron with an NSRI. While we found no similar study on utilization of saffron for treatment of chronic pain conditions, critical appraisal of this study is plausible in the context of previous trials which compared the efficacy of saffron with antidepressants for treatment of depression. 

In this study, we found that mean score changes from baseline to the endpoint were not significantly different in terms of the scales measuring mental or physical symptoms of fibromyalgia, between saffron and duloxetine arms. This finding implies comparable efficacy of the two medications. In previous studies, saffron exhibited comparable efficacy to imipramine, citalopram, and fluoxetine in MDD (Akhondzadeh et al., 2004 ▶; Noorbala et al., 2005 ▶; Basti et al., 2007 ▶; Ghajar et al., 2016 ▶), to fluoxetine in mild/moderate depression after percutaneous coronary intervention (Shahmansouri et al., 2014 ▶), and to fluoxetine in post-partum depression (Kashani et al., 2017 ▶). 

As a medicinal plant with different active constituents, potential mechanisms underlying saffron effects on FM involve multiple neuroendocrine systems as well as anti-inflammatory and antioxidant effects which have been discussed in detail (Lopresti et al., 2014 ▶; Khazdair et al., 2015 ▶; Moshiri et al., 2015 ▶). In brief, in the central nervous system, saffron increases serotonin, norepinephrine, and dopamine (Sarris et al., 2011 ▶; Khazdair et al., 2015 ▶), which are all lowered in FM (Becker and Schweinhardt, 2012 ▶; Thiagarajah et al., 2014 ▶). Anti-inflammatory and antioxidant effects of saffron in the CNS are also documented (Lopresti and Drummond, 2014 ▶; Khazdair et al., 2015 ▶). On the other hand, there is evidence for heightened inflammatory state and increased oxidative stress in FM (Cordero et al., 2010 ▶; Meeus et al., 2013 ▶). Cumulative effects of saffron caused by all its active constituents through different mechanisms, may be responsible for its effects on FM which were comparable to those of duloxetine. Taking into account that most of previous studies on saffron’s mechanism of action, was carried out in animal models, and that active constituents of saffron may have interactions (e.g. synergism) in-terms of neurobiological effects (Lopresti and Drummond, 2014 ▶), it is necessary to focus on elucidating the exact mechanisms of action of saffron constituents in the CNS in patients with FM and depression, which in turn will provide firm grounds for wider applications of this compound in clinical settings. 

Similar to previous clinical trials that compared saffron to anti-depressants, this study was subjected to some limitations which cumulatively imply caution in terms of interpretation and generalizability of the results. First, all studies were designed as superiority trials and lacked a placebo arm. In fact, it is important to consider that when comparing a novel test drug to an approved medication, the proper design is non-inferiority rather than superiority (Schumi and Wittes, 2011 ▶). However, non-inferiority trials usually require very large sample sizes and are subjected to additional complexities which make them impractical in many instances (Schumi and Wittes, 2011 ▶). The alternative method is to include a placebo arm in a superiority trial which provides a mean for real-time comparison of both drugs to placebo and to each other in order to verify that both the test and the standard drugs are superior to placebo while they may be non-superior to each other. In this way, strong implication is provided for non-inferiority as well. This study and previous trials of saffron vs. antidepressants lacked a placebo arm. However, there had been prior placebo-controlled trials of saffron in both clinical and subclinical depression with dramatic and significant differences between placebo and saffron, which make this defect less detrimental. Second, because of the relatively small sample size of the study, we were not able to perform a non-inferiority analysis after approval of non-superiority. Meanwhile, we calculated the sample size considering a power of 80% for this trial as the first trial studying the effects of saffron in FM. Third, using a low fixed dose of saffron for a short period of time (i.e. 6-8 weeks) in previous clinical trials as well as this trial, provided insufficient information for long-term adverse effects of saffron compared to antidepressants. However, saffron has been proven to have a good safety profile with a wide therapeutic window in almost all previous clinical studies, and has been widely used as a food additive in Iran. Fourth, we did not take into account the proposed subgrouping of FM (Luciano et al., 2016 ▶). In more details, it has been suggested that limited efficacy of therapeutics for treatment of FM may be due to the heterogeneity of the condition, and some subgroups of FM may show better outcomes following administration of certain treatments (Lawson, 2016 ▶). Six, due to budgetary and executive limitations, aside from paraclinical evaluations that concerned safety issues, we assessed no biological markers of FM; this is highly suggested to be addressed in future as it may lead to discover saffron’s mechanisms of action . 

In conclusion, this study provided preliminary evidence on comparable efficacy of saffron and duloxetine in treatment of FM. Future comprehensive clinical trials are warranted considering the limitations of this study. It is also noteworthy that though drug monotherapy is the favored method of treatment in many clinical conditions, FM as a multidimensional disorder with highly variable comorbidities, may require combination therapy to achieve dramatic improvement in symptoms. In this manner, saffron as an adjunctive treatment to the currently approved medications or to non-pharmacological interventions, would be an interesting topic for further investigations.

## References

[B1] Ablin JN, Häuser W (2016). Fibromyalgia syndrome: novel therapeutic targets. Pain Manag.

[B2] Akhondzadeh S, Fallah-Pour H, Afkham K, Jamshidi A-H, Khalighi-Cigaroudi F (2004). Comparison of Crocus sativus L and imipramine in the treatment of mild to moderate depression: a pilot double-blind randomized trial [ISRCTN45683816]. BMC Complement Altern Med.

[B3] American Psychiatric Association (2000). DSM-IV-TR: Diagnostic and statistical manual of mental disorders, text revision.

[B4] Amin B, Hosseini S, Hosseinzadeh H (2017). Enhancement of Antinociceptive Effect by Co-administration of Amitriptyline and Crocus sativus in a Rat Model of Neuropathic Pain. Iran J Pharm Res.

[B5] Asadabadi M, Mohammadi MR, Ghanizadeh A, Modabbernia A, Ashrafi M, Hassanzadeh E, Forghani S, Akhondzadeh S (2013). Celecoxib as adjunctive treatment to risperidone in children with autistic disorder: a randomized, double-blind, placebo-controlled trial. Psychopharmacol.

[B6] Basti AA, Moshiri E, Noorbala A-A, Jamshidi A-H, Abbasi SH, Akhondzadeh S (2007). Comparison of petal of Crocus sativus L and fluoxetine in the treatment of depressed outpatients: a pilot double-blind randomized trial. Prog Neuropsychopharmacol Biol Psychiatry.

[B7] Becker S, Schweinhardt P (2012). Dysfunctional neurotransmitter systems in fibromyalgia, their role in central stress circuitry and pharmacological actions on these systems. Pain Res Treat.

[B8] Burckhardt CS, Clark SR, Bennett RM (1991). The fibromyalgia impact questionnaire: development and validation. J Rheumatol.

[B9] Calandre EP, Rico-Villademoros F, Slim M (2015). An update on pharmacotherapy for the treatment of fibromyalgia. Expert Opin Pharmacother.

[B10] Carlsson AM (1983). Assessment of chronic pain I Aspects of the reliability and validity of the visual analogue scale. Pain.

[B11] Chinn S, Caldwell W, Gritsenko K (2016). Fibromyalgia pathogenesis and treatment options update. Curr Pain Headache Rep.

[B12] Cleeland CS, Ryan KM (1994). The Brief Pain Inventory. Ann Acad Med Singapore.

[B13] Cordero MD, de Miguel M, Carmona-López I, Bonal P, Campa F, Moreno-Fernández AM (2010). Oxidative stress and mitochondrial dysfunction in fibromyalgia. Neuro Endocrinol Lett.

[B14] De Souza Nascimento S, DeSantana JM, Nampo FK, Ribeiro ÊA, da Silva DL, Araújo-Júnior JX, da Silva Almeida JR, Bonjardim LR, de Souza Araújo AA, Quintans-Júnior LJ (2013). Efficacy and safety of medicinal plants or related natural products for fibromyalgia: a systematic review. Evid Based Complement Alternat Med.

[B15] Ghajar A, Neishabouri SM, Velayati N, Jahangard L, Matinnia N, Haghighi M, Ghaleiha A, Afarideh M, Salimi S, Meysamie A, Akhondzadeh S (2017). Crocus sativus L versus Citalopram in the treatment of major depressive disorder with anxious distress: A double-blind controlled clinical trial. Pharmacopsychiatry.

[B16] Ghavidel-Parsa B, Amir Maafi A, Haghdoost A, Arabi Y, Khojamli M, Chatrnour G, Bidari A (2014). The validity and reliability of the Persian version of the Revised Fibromyalgia Impact Questionnaire. Rheumatol Int.

[B17] Ghavidel-Parsa B, Bidari A, Amir Maafi A, Ghalebaghi B (2015). The iceberg nature of fibromyalgia burden: the clinical and economic aspects. Korean J Pain.

[B18] Gracely RH, Ceko M, Bushnell MC (2012). Fibromyalgia and depression. Pain Res Treat.

[B19] Hamilton M (1960). A rating scale for depression. J Neurol Neurosurg Psychiatry.

[B20] Hausenblas HA, Saha D, Dubyak PJ, Anton SD (2013). Saffron (Crocus sativus L) and major depressive disorder: a meta-analysis of randomized clinical trials. J Integr Med.

[B21] Häuser W, Petzke F, Sommer C (2010). Comparative efficacy and harms of duloxetine, milnacipran, and pregabalin in fibromyalgia syndrome. J Pain.

[B22] Häuser W, Urrútia G, Tort S, Üçeyler N, Walitt B (2013). Serotonin and noradrenaline reuptake inhibitors (SNRIs) for fibromyalgia syndrome. Cochrane Database Syst Rev.

[B23] Häuser W, Henningsen P (2014a). Fibromyalgia syndrome: a somatoform disorder?. Eur J Pain.

[B24] Häuser W, Walitt B, Fitzcharles M-A, Sommer C (2014b). Review of pharmacological therapies in fibromyalgia syndrome. Arthritis Res Ther.

[B25] Hewlett S, Dures E, Almeida C (2011). Measures of fatigue: Bristol Rheumatoid Arthritis Fatigue Multi‐Dimensional Questionnaire (BRAF MDQ), Bristol Rheumatoid Arthritis Fatigue Numerical Rating Scales (BRAF NRS) for Severity, Effect, and Coping, Chalder Fatigue Questionnaire (CFQ), Checklist Individual Strength (CIS20R and CIS8R), Fatigue Severity Scale (FSS), Functional Assessment Chronic Illness Therapy (Fatigue)(FACIT‐F), Multi‐Dimensional Assessment of Fatigue (MAF), Multi‐Dimensional Fatigue Inventory (MFI), Pediatric Quality Of Life (PedsQL) Multi‐Dimensional Fatigue Scale, Profile of Fatigue (ProF), Short Form 36 Vitality Subscale (SF‐36 VT), and Visual Analog Scales (VAS). Arthritis Care Res.

[B26] Kashani L, Raisi F, Saroukhani S, Sohrabi H, Modabbernia A, Nasehi AA, Jamshidi A, Ashrafi M, Mansouri P, Ghaeli P, Akhondzadeh S (2013). Saffron for treatment of fluoxetine‐induced sexual dysfunction in women: randomized double‐blind placebo‐controlled study. Hum Psychopharmacol.

[B27] Kashani L, Eslatmanesh S, Saedi N, Niroomand N, Ebrahimi M, Hosseinian M, Foroughifar T, Salimi S, Akhondzadeh S (2017). Comparison of Saffron versus Fluoxetine in Treatment of Mild to Moderate Postpartum Depression: A Double-Blind, Randomized Clinical Trial. Pharmacopsychiatry.

[B28] Kashani L, Eslatmanesh S, Eftekhari F, Salimi S, Foroughifar T, Etesam F, Safiaghdam H, Moazen-Zadeh E, Akhondzadeh S (2018). Efficacy of Crocus sativus (saffron) in treatment of major depressive disorder associated with post-menopausal hot flashes: a double-blind, randomized, placebo-controlled trial. Arch Gynecol Obstet.

[B29] Khazdair MR, Boskabady MH, Hosseini M, Rezaee R, Tsatsakis AM (2015). The effects of Crocus sativus (saffron) and its constituents on nervous system: A review. Avicenna J Phytomed.

[B30] Khodaie-Ardakani MR, Mirshafiee O, Farokhnia M, Tajdini M, Hosseini SM, Modabbernia A, Rezaei F, Salehi B, Yekehtaz H, Ashrafi M, Tabrizi M, Akhondzadeh S (2014). Minocycline add-on to risperidone for treatment of negative symptoms in patients with stable schizophrenia: randomized double-blind placebo-controlled study. Psychiatry Res.

[B31] Lauche R, Cramer H, Häuser W, Dobos G, Langhorst J (2015). A systematic overview of reviews for complementary and alternative therapies in the treatment of the fibromyalgia syndrome. Evid Based Complement Alternat Med.

[B32] Lawson K (2016). Potential drug therapies for the treatment of fibromyalgia. Expert Opin Investig Drugs.

[B33] Lopresti AL, Drummond PD (2014). Saffron (Crocus sativus) for depression: a systematic review of clinical studies and examination of underlying antidepressant mechanisms of action. Hum Psychopharmacol.

[B34] Luciano JV, Forero CG, Cerdà-Lafont M, Peñarrubia-María MT, Fernández-Vergel R, Cuesta-Vargas AI, Ruíz JM, Rozadilla-Sacanell A, Sirvent-Alierta E, Santo-Panero P, García-Campayo J (2016). Functional Status, Quality of Life, and Costs Associated With Fibromyalgia Subgroups. Clin J Pain.

[B35] Maletic V, Raison CL (2009). Neurobiology of depression, fibromyalgia and neuropathic pain. Front Biosci.

[B36] Mazidi M, Shemshian M, Mousavi SH, Norouzy A, Kermani T, Moghiman T, Sadeghi A, Mokhber N, Ghayour-Mobarhan M, Ferns GA (2016). A double-blind, randomized and placebo-controlled trial of Saffron (Crocus sativus L) in the treatment of anxiety and depression. J Complement Integr Med.

[B37] Meeus M, Nijs J, Hermans L, Goubert D, Calders P (2013). The role of mitochondrial dysfunctions due to oxidative and nitrosative stress in the chronic pain or chronic fatigue syndromes and fibromyalgia patients: peripheral and central mechanisms as therapeutic targets?. Expert Opin Ther Targets.

[B38] Milajerdi A, Bitarafan V, Mahmoudi M (2015). A review on the effects of saffron extract and its constituents on factors related to neurologic, cardiovascular and gastrointestinal diseases. J Med Plants.

[B39] Moazen-Zadeh E, Abbasi SH, Safi-Aghdam H, Shahmansouri N, Arjmandi-Beglar A, Hajhosseinn Talasaz A, Salehiomran A, Forghani S, Akhondzadeh S (2018). Effects of Saffron on Cognition, Anxiety, and Depression in Patients Undergoing Coronary Artery Bypass Grafting: A Randomized Double-Blind Placebo-Controlled Trial. J Altern Complement Med.

[B40] Modabbernia A, Sohrabi H, Nasehi AA, Raisi F, Saroukhani S, Jamshidi A, Tabrizi M, Ashrafi M, Akhondzadeh S (2012). Effect of saffron on fluoxetine-induced sexual impairment in men: randomized double-blind placebo-controlled trial. Psychopharmacol.

[B41] Moshiri M, Vahabzadeh M, Hosseinzadeh H (2015). Clinical applications of saffron (Crocus sativus) and its constituents: a review. Drug Res.

[B42] Noorbala AA, Akhondzadeh S, Tahmacebi-Pour N, Jamshidi AH (2005). Hydro-alcoholic extract of Crocus sativus L versus fluoxetine in the treatment of mild to moderate depression: a double-blind, randomized pilot trial. J Ethnopharmacol.

[B43] Pae CU, Luyten P, Marks DM, Han C, Park SH, Patkar AA, Masand PS, Van Houdenhove B (2008). The relationship between fibromyalgia and major depressive disorder: a comprehensive review. Curr Med Res Opin.

[B44] Rezaei F, Mohammad-Karimi M, Seddighi S, Modabbernia A, Ashrafi M, Salehi B, Hammidi S, Motasami H, Hajiaghaee R, Tabrizi M, Akhondzadeh S (2013). Memantine add-on to risperidone for treatment of negative symptoms in patients with stable schizophrenia: randomized, double-blind, placebo-controlled study. J Clin Psychopharmacol.

[B45] Queiroz LP (2013). Worldwide epidemiology of fibromyalgia. Curr Pain Headache Rep.

[B46] Sadreddini S, Molaeefard M, Noshad H, Ardalan M, Asadi A (2008). Efficacy of Raloxifen in treatment of fibromyalgia in menopausal women. Eur J Intern Med.

[B47] Safakhah HA, Taghavi T, Rashidy-Pour A, Vafaei AA, Sokhanvar M, Mohebbi N, Rezaei-Tavirani M Effects of saffron (Crocus sativus L) stigma extract and its active constituent crocin on neuropathic pain responses in a rat model of chronic constriction injury. Iran J Pharm Res.

[B48] Sarris J, Panossian A, Schweitzer I, Stough C, Scholey A (2011). Herbal medicine for depression, anxiety and insomnia: a review of psychopharmacology and clinical evidence. Eur Neuropsychopharmacol.

[B49] Schmidt-Wilcke T, Clauw DJ (2011). Fibromyalgia: from pathophysiology to therapy. Nat Rev Rheumatol.

[B50] Schumi J, Wittes JT (2011). Through the looking glass: understanding non-inferiority. Trials.

[B51] Shahmansouri N, Farokhnia M, Abbasi SH, Kassaian SE, Tafti AA, Gougol A, Yekehtaz H, Forghani S, Mahmoodian M, Saroukhani S, Arjmandi-Beglar A (2014). A randomized, double-blind, clinical trial comparing the efficacy and safety of Crocus sativus L with fluoxetine for improving mild to moderate depression in post percutaneous coronary intervention patients. J Affect Disord.

[B52] Sluka KA, Clauw DJ (2016). Neurobiology of fibromyalgia and chronic widespread pain. Neurosci.

[B53] Theoharides TC, Tsilioni I, Arbetman L, Panagiotidou S, Stewart JM, Gleason RM, Russell IJ (2015). Fibromyalgia syndrome in need of effective treatments. J Pharmacol Exp Ther.

[B54] Thiagarajah AS, Guymer EK, Leech M, Littlejohn GO (2014). The relationship between fibromyalgia, stress and depression. Int J Clin Rheumatol.

[B55] Üçeyler N, Sommer C, Walitt B, Häuser W (2013). Anticonvulsants for fibromyalgia. Cochrane Database Syst Rev.

[B56] Wolfe F, Clauw DJ, Fitzcharles MA, Goldenberg DL, Katz RS, Mease P, Russell AS, Russell IJ, Winfield JB, Yunus MB (2010). The American College of Rheumatology preliminary diagnostic criteria for fibromyalgia and measurement of symptom severity. Arthritis Care Res.

[B57] Zigmond AS, Snaith RP (1983). The hospital anxiety and depression scale. Acta Psychiatr Scand.

